# Integrated oxidative stress score for predicting prognosis in stage III gastric cancer undergoing surgery

**DOI:** 10.3389/pore.2023.1610897

**Published:** 2023-06-02

**Authors:** Yu-hang Liu, Rui Meng, Bing Zhu, Qi-qi Zhan, Xin Yang, Guan-yi Ding, Chun-liang Jia, Qian-yu Liu, Wei-guo Xu

**Affiliations:** ^1^ School of Clinical Medicine, North China University of Science and Technology, Tangshan, China; ^2^ Department of Emergency Intensive Care Unit, Yangpu Hospital, Tongji University, Shanghai, China; ^3^ Tangshan Gongren Hospital, Tangshan, China; ^4^ North China University of Science and Technology Affiliated Hospital, Tangshan, China; ^5^ Tangshan People’s Hospital, Tangshan, China; ^6^ Department of Gastrointestinal Surgery, China Hospital Medical Sciences, Shenzhen, China

**Keywords:** gastric cancer, oxidative stress, albumin, bilirubin, blood urea nitrogen

## Abstract

**Objective:** This study aimed to develop a novel scoring system, named the integrated oxidative stress score (IOSS), based on oxidative stress indices to predict the prognosis in stage III gastric cancer.

**Methods:** Retrospective analysis of stage III gastric cancer patients who were operated on between January 2014 and December 2016 were enrolled into this research. IOSS is a comprehensive index based on an achievable oxidative stress index, comprising albumin, blood urea nitrogen, and direct bilirubin. The patients were divided according to receiver operating characteristic curve into two groups of low IOSS (IOSS ≤ 2.00) and high IOSS (IOSS > 2.00). The grouping variable was performed by Chi-square test or Fisher’s precision probability test. The continuous variables were evaluated by t-test. The disease free survival (DFS) and overall survival (OS) were performed by Kaplan-Meier and Log-Rank tests. Univariate Cox proportional hazards regression models and stepwise multivariate Cox proportional hazards regression analysis were determined to appraise the potential prognostic factors for DFS and OS. A nomogram of the potential prognostic factors by the multivariate analysis for DFS and OS was established with R software. In order to assess the accuracy of the nomogram in forecasting prognosis, the calibration curve and decision curve analysis were produced, contrasting the observed outcomes with the predicted outcomes.

**Results:** The IOSS was significantly correlated with the DFS and OS, and was a potential prognostic factor in patients with stage III gastric cancer. Patients with low IOSS had longer survival (DFS: χ^2^ = 6.632, *p* = 0.010; OS: χ^2^ = 6.519, *p* = 0.011), and higher survival rates. According to the univariate and multivariate analyses, the IOSS was a potential prognostic factor. The nomograms were conducted on the potential prognostic factors to improve the correctness of survival prediction and evaluate the prognosis in stage III gastric cancer patients. The calibration curve indicated a good agreement in 1-, 3-, 5-year lifetime rates. The decision curve analysis indicated that the nomogram’s predictive clinical utility for clinical decision was better than IOSS.

**Conclusion:** IOSS is a nonspecific tumor predictor based on available oxidative stress index, and low IOSS is found to be a vigorous factor of better prognosis in stage III gastric cancer.

## Introduction

Gastric cancer ranked sixth among the global cancer burdens in both females and males in 2020, and remains one of the prime causes of cancer-related morbidity and cancer-related death in the world [1, 2]. The incidence of gastric cancer is highest in Eastern Asia, such as South Korea and China, and is largely dependent on *Helicobacter pylori* infection, co-infection by Epstein-Barr virus (EBV), gastroesophageal reflux disease, and obesity [3, 4]. Despite changes in dietary habits and improvements in living conditions, the mortality of gastric cancer is still high in recent decades [5, 6]. Currently, the surgical operation remains the primary curative therapy for early-stage gastric cancer [7]. Complex treatment, such as chemotherapy, radiotherapy, targeted therapy and immunotherapy, is used for gastric cancer or metastatic disease [8]. Although these treatments have significantly improved the overall survival of gastric cancer patients, unfortunately, the long-term survival rate is unsatisfactory. Approximately 70% of gastric cancer patients have distant metastasis or recurrence within 5 years [9]. As a result of geographical, genetic, and dietary differences, predicting the survival and prognosis of gastric cancer patients can be challenging [10, 11]. However, the precise prediction of prognosis is crucial for treatment election and doctor-patient communication. Consequently, it is very important to look for the potential prognostic factor for gastric cancer.

Previous studies have indicated that systemic inflammation, malnutrition, and TNM staging system can usually predict survival of gastric cancer patients [12–14]. Nevertheless, gastric cancer is a heterogeneous disorder. Even if the patients are at the same stage of disease, they may have different prognoses [15]. The present TNM staging system is uniform and simple, and does not consider several noteworthy variables that may influence the survival of gastric cancer patients, comprising clinicopathological characteristics and adjuvant treatment. Hence, the prognosis of gastric cancer patients cannot be accurately predicted. Currently, the long-term prognosis of gastric cancer is still poor, and warranting an effective and reasonable predictive model is needed to evaluate the long-term prognosis of gastric cancer patients.

Oxidative stress, which is defined as an imbalance between reactive metabolites and free radicals, interacts with the occurrence, development, and progression of malignant tumors [16, 17]. Previous studies have shown that foods such as vegetables, fruits, seeds, and whole grains can counteract oxidative stress and inflammation, and may also be beneficial to cancer patients [18]. Henriksen HB et al. found that a healthy diet played a critical role in suppressing oxidative stress and inflammation, affecting survival time and outcome in colorectal cancer patients [19]. Moreover, the progression-related hub oxidative stress genes were confirmed to be conspicuously related to the progression of gastric cancer according to the weighted gene co-expression network analysis (WGCNA) and Gene Expression Omnibus (GEO) database [20]. Early assessment of oxidative stress for patients with gastric cancer can ameliorate the clinical outcomes. One study has indicated that the oxidative stress genes, such as NQO1 and PON1, were the noteworthy prognostic factors in metastatic gastric cancer patients treated with chemotherapy; and the oxidative stress-related genetic variants may facilitate optimization of individualized chemotherapy in clinical practice [21]. Another study indicated that the CRC-Integrated Oxidative Stress Score (CIOSS) based on the combination of available oxidative stress indices (albumin, direct bilirubin, and blood urea nitrogen) was significantly associated with survival in CRC patients; and the CIOSS was a powerful indicator of poor prognosis in CRC patients [22]. Taking into consideration the important function of oxidative stress in the development of gastric cancer, we tried to study the potential prognostic implication of oxidative stress correlated indicators. In the current study, we validated a gastric cancer Integrated Oxidative Stress Score (IOSS), and aimed to determine the prognostic significance of IOSS in stage III gastric cancer. Furthermore, we compared the relevance between IOSS and clinicopathological characteristics.

## Materials and methods

### Patients and study design

198 patients with gastric cancer who were treated at the North China University of Science and Technology Affiliated Hospital between January 2014 and December 2016 were brought into this study. We excluded patients with clinical evidence of inflammatory, infectious, and hematological diseases, and without complete clinical data. This study was authorized by the Ethics Review Committee of the North China University of Science and Technology Affiliated Hospital, and it meets the criteria of the Declaration of Helsinki and its later revised drafts. All enrolled patients signed the agreement, and individual patient information has been protected and not been shown.

The inclusion criteria were as follows: 1) confirmed gastric cancer by histopathology; 2) received primary tumor resection without evidence of distant metastasis; 3) without inflammatory, infectious, and hematological diseases; 4) availability of whole clinical and pathological data. Participants were considered ineligible if they were gastric cancer patients who had: 1) multiple primary malignant tumors or blood disease; 2) presence of kidney dysfunction, metabolic diseases, or cardiovascular disease; 3) received anti-inflammatory drugs prior to surgery; 4) no follow-up information.

### Integrated oxidative stress score (IOSS)

The blood routine and biochemical detection were performed from the first day of admission for gastric cancer patients. In the previous study, the oxidative stress indicators (albumin, bilirubin, and blood urea nitrogen) were found to be independent risk factors and significantly correlated with OS or DFS by performing Cox regression in CRC patients [22]. The Integrated Oxidative Stress Score (IOSS) in our study comprised albumin (ALB), blood urea nitrogen (BUN), and direct bilirubin (DBIL). The IOSS was calculated as follows: 0.074 × albumin (g/L)–0.094 × bilirubin (μmol/L)–0.099 × blood urea nitrogen (mmol/L), according to the previous study [22].

### Follow-up

The follow-up data were gathered through telephone interviews or inpatient and outpatient check-ups, and were obtained every 3 months. In this study, disease free survival (DFS) was defined as the time from operation to local recurrence of tumor, or death or last follow-up. Overall survival (OS) was defined as the time from operation to death or last follow-up. The last follow-up was assessed in June 2022.

### Statistical analysis

SPSS 22.0 (SPSS Inc., Chicago, IL, United States) and R 4.1.2 software (Institute for Statistics and Mathematics, Vienna, Austria) were used to perform all statistical analyses. The optimal cutoff value for IOSS was performed by the receiver operating characteristic (ROC) curve analysis. The nominal variables were assessed by Chi-square test or Fisher’s precision probability test. The continuous variables were evaluated by t-test. The DFS and OS time were performed by Kaplan-Meier method and Log-Rank test. Univariate and multivariate Cox proportional hazards regression models were determined to evaluate the potential prognostic factors for DFS and OS. The nomogram model of the potential prognostic factors by the multivariate analysis for DFS and OS were established with R software. In order to assess the precision of the nomogram model in forecasting prognosis, the calibration curve and decision curve analysis were generated comparing the observations with the predictions. *p* values of <0.05 were considered statistically significant.

## Results

### Study population

In total, 198 patients with gastric cancer from North China University of Science and Technology Affiliated Hospital were included in this research. There were 128 males (64.6%) and 70 females (35.4%). The average age was 59.37 ± 11.10 years, and ranged from 30 to 80 years. The optimal cutoff value for IOSS was performed by ROC, and the value was 2.00. Based on the optimal cutoff value, all cases were divided into two groups: low IOSS group (IOSS ≤ 2.00) and high IOSS group (IOSS > 2.00). The clinical characteristics of gastric cancer patients were given in Table 1. Compared to the patients’ clinical and histopathological features, IOSS indicated significant relationship with age, radical resection, and tumor size (*p* < 0.05).

**TABLE 1:** Association of IOSS and patient characteristics.

Characteristics	Level	Low IOSS	High IOSS	*p*
*n*		91	107	
Sex	Male	64 (70.3)	64 (59.8)	0.163
	Female	27 (29.7)	43 (40.2)	
Age	≤60	33 (36.3)	64 (59.8)	**0.002**
	>60	58 (63.7)	43 (40.2)	
Profession	White collar workers	21 (23.1)	18 (16.8)	0.356
	Blue collar workers	70 (76.9)	89 (83.2)	
BMI	≤22.0	49 (53.8)	46 (43.0)	0.167
	>22.0	42 (46.2)	61 (57.0)	
Radical resection	R0	69 (75.8)	93 (86.9)	**<0.001**
	R1	20 (22.0)	3 (2.8)	
	R2	2 (2.2)	11 (10.3)	
Type of surgery	distal gastrectomy	77 (84.6)	81 (75.7)	0.296
	proximal gastrectomy	3 (3.3)	6 (5.6)	
	total gastrectomy	11 (12.1)	20 (18.7)	
Primary tumor site	upper 1/3	8 (8.8)	10 (9.3)	0.456
	middle 1/3	8 (8.8)	13 (12.1)	
	low 1/3	66 (72.5)	67 (62.6)	
	whole	9 (9.9)	17 (15.9)	
Borrmann type	Borrmann 0	0 (0.0)	2 (1.9)	0.189
	Borrmann I	4 (4.4)	3 (2.8)	
	Borrmann II	19 (20.9)	18 (16.8)	
	Borrmann III	63 (69.2)	69 (64.5)	
	Borrmann IV	3 (3.3)	13 (12.1)	
	Borrmann V	2 (2.2)	2 (1.9)	
Tumor size	≤20 mm	14 (15.4)	21 (19.6)	**0.020**
	>20 and <50 mm	51 (56.0)	39 (36.4)	
	≥50 mm	26 (28.6)	47 (43.9)	
Differentiation	poorly differentiated	38 (41.8)	61 (57.0)	0.101
	moderately differentiated	52 (57.1)	45 (42.1)	
	well differentiated	1 (1.1)	1 (0.9)	
Pathology	adenocarcinoma	37 (40.7)	33 (30.8)	0.314
	mucinous carcinoma	4 (4.4)	4 (3.7)	
	signet ring cell carcinoma	2 (2.2)	6 (5.6)	
	mixed carcinoma	48 (52.7)	62 (57.9)	
	others	0 (0.0)	2 (1.9)	
pTNM stage	IIIA	35 (38.5)	40 (37.4)	0.956
	IIIB	38 (41.8)	44 (41.1)	
	IIIC	18 (19.8)	23 (21.5)	
pT stage	T2	1 (1.1)	2 (1.9)	0.437
	T3	53 (58.2)	50 (46.7)	
	T4a	29 (31.9)	42 (39.3)	
	T4b	8 (8.8)	13 (12.1)	
pN stage	N0	1 (1.1)	6 (5.6)	0.389
	N1	11 (12.1)	12 (11.2)	
	N2	32 (35.2)	43 (40.2)	
	N3a	33 (36.3)	34 (31.8)	
	N3b	14 (15.4)	12 (11.2)	
Lauren type	Intestinal	43 (47.3)	44 (41.1)	0.687
	Diffuse	22 (24.2)	29 (27.1)	
	Mixed	26 (28.6)	34 (31.8)	
Postoperative chemotherapy	No	39 (42.9)	55 (51.4)	0.290
	Yes	52 (57.1)	52 (48.6)	

Abbreviation: BMI, body mass index; TNM, tumor node metastasis. Bold values are primarily used to identify meaningful variables.

### IOSS and blood parameters

The blood routine and biochemical parameters were enrolled into this research and the median values were used to group these indicators. Table 2 has shown the associations of IOSS and the blood routine and biochemical parameters. According to the patient characteristics of blood routine and biochemical parameters, IOSS indicated a significant relationship with total protein (TP), albumin (ALB), prealbumin (PALB), blood urea nitrogen (BUN), triglyceride (TRIG), hemoglobin (Hb), and red blood cell R) (*p* < 0.05).

**TABLE 2:** Association between IOSS and blood routine and biochemical parameters.

Parameters	Level	Low IOSS	High IOSS	*p*
*n*		91	107	
Alanine aminotransferase	≤18.0	50 (54.9)	47 (43.9)	0.161
	>18.0	41 (45.1)	60 (56.1)	
Aspartate aminotransferase	≤20.0	39 (42.9)	53 (49.5)	0.426
	>20.0	52 (57.1)	54 (50.5)	
γ-glutamyl transferase	≤14.0	47 (51.6)	50 (46.7)	0.584
	>14.0	44 (48.4)	57 (53.3)	
Lactate dehydrogenase	≤165.0	46 (50.5)	52 (48.6)	0.896
	>165.0	45 (49.5)	55 (51.4)	
Total bilirubin	≤10.2	44 (48.4)	54 (50.5)	0.878
	>10.2	47 (51.6)	53 (49.5)	
Direct bilirubin	≤3.8	41 (45.1)	57 (53.3)	0.313
	>3.8	50 (54.9)	50 (46.7)	
Indirect bilirubin	≤6.5	46 (50.5)	53 (49.5)	1.000
	>6.5	45 (49.5)	54 (50.5)	
Total protein	≤65.0	65 (71.4)	33 (30.8)	**<0.001**
	>65.0	26 (28.6)	74 (69.2)	
Albumin	≤40.0	66 (72.5)	23 (21.5)	**<0.001**
	>40.0	25 (27.5)	84 (78.5)	
Globulin	≤25.0	44 (48.4)	41 (38.3)	0.201
	>25.0	47 (51.6)	66 (61.7)	
Albumin/Globulin (A/G)	≤1.5	49 (53.8)	47 (43.9)	0.212
	>1.5	42 (46.2)	60 (56.1)	
Prealbumin	≤230.0	58 (63.7)	40 (37.4)	**<0.001**
	>230.0	33 (36.3)	67 (62.6)	
Blood urea nitrogen	≤5.5	31 (34.1)	61 (57.0)	**0.002**
	>5.5	60 (65.9)	46 (43.0)	
Alkaline phosphatase	≤73.0	43 (47.3)	55 (51.4)	0.660
	>73.0	48 (52.7)	52 (48.6)	
Glucose	≤5.1	48 (52.7)	46 (43.0)	0.220
	>5.1	43 (47.3)	61 (57.0)	
Cholesterol	≤4.2	51 (56.0)	46 (43.0)	0.091
	>4.2	40 (44.0)	61 (57.0)	
Triglyceride	≤1.1	55 (60.4)	42 (39.3)	**0.005**
	>1.1	36 (39.6)	65 (60.7)	
ABO blood type	A	28 (30.8)	46 (43.0)	0.229
	B	37 (40.7)	30 (28.0)	
	O	19 (20.9)	22 (20.6)	
	AB	7 (7.7)	9 (8.4)	
White blood cell	≤6.5	50 (54.9)	48 (44.9)	0.203
	>6.5	41 (45.1)	59 (55.1)	
Neutrophils	≤3.7	46 (50.5)	53 (49.5)	1.000
	>3.7	45 (49.5)	54 (50.5)	
Lymphocyte	≤1.9	47 (51.6)	48 (44.9)	0.418
	>1.9	44 (48.4)	59 (55.1)	
Monocyte	≤0.5	48 (52.7)	50 (46.7)	0.483
	>0.5	43 (47.3)	57 (53.3)	
Eosinophils	≤0.1	43 (47.3)	55 (51.4)	0.660
	>0.1	48 (52.7)	52 (48.6)	
Basophil	≤0.03	37 (40.7)	50 (46.7)	0.475
	>0.03	54 (59.3)	57 (53.3)	
Hemoglobin	≤135	61 (67.0)	38 (35.5)	**<0.001**
	>135	30 (33.0)	69 (64.5)	
Red blood cell	≤4.3	56 (61.5)	41 (38.3)	**0.002**
	>4.3	35 (38.5)	66 (61.7)	
Platelet	≤255.0	48 (52.7)	50 (46.7)	0.483
	>255.0	43 (47.3)	57 (53.3)	
International normalized ratio	≤1.0	42 (46.2)	52 (48.6)	0.841
	>1.0	49 (53.8)	55 (51.4)	
Fibrinogen	≤3.0	43 (47.3)	57 (53.3)	0.483
	>3.0	48 (52.7)	50 (46.7)	
Carcinoembryonic antigen	≤2.1	41 (45.1)	58 (54.2)	0.254
	>2.1	50 (54.9)	49 (45.8)	
Alpha fetoprotein	≤2.7	47 (51.6)	51 (47.7)	0.677
	>2.7	44 (48.4)	56 (52.3)	
Carbohydrate antigen199	≤12.8	48 (52.7)	53 (49.5)	0.758
	>12.8	43 (47.3)	54 (50.5)	
Carbohydrate antigen724	≤2.5	49 (53.8)	51 (47.7)	0.469
	>2.5	42 (46.2)	56 (52.3)	

Abbreviation: ALT, alanine aminotransferase; AST, aspartate aminotransferase; GGT, γ-glutamyl transferase; LDH, lactate dehydrogenase; TBIL, total bilirubin; DBIL, direct bilirubin; IDBIL, indirect bilirubin; TP, total protein; ALB, albumin; GLOB, globulin; PALB, prealbumin; A/G, ALB/GLOB; BUN, blood urea nitrogen; ALP, alkaline phosphatase; Glu, glucose; CHOL, cholesterol; TRIG, triglyceride; W, white blood cell; N, neutrophils; L, lymphocyte; M, monocyte; E, eosinophils; B, basophil; Hb, hemoglobin; R, red blood cell; P, platelet; INR, international normalized ratio; FIB, fibrinogen; CEA, carcinoembryonic antigen; AFP, alpha fetoprotein; CA199, Carbohydrate antigen199; CA724, Carbohydrate antigen724. Bold values are primarily used to identify meaningful variables.

### IOSS and pathological characteristics

In this study, human epidermal growth factor receptor-2 (HER-2) positive means IHC (+++) or IHC (++) and FISH (+). We further investigated the association between IOSS and pathological characteristics. The results displayed that IOSS was related to the HER2 and perineural invasion (PNI) (*p* < 0.05). However, IOSS was not significantly correlated with lymphatic vessel invasion (LVI) (*p* > 0.05). Table 3 has shown the relationships of IOSS and the pathological characteristics parameters.

**TABLE 3:** The relationships of IOSS and the pathological characteristics parameters.

Parameters	Level	Low IOSS	High IOSS	*p*
*n*		91	107	
TLN	≤25	40 (44.0)	55 (51.4)	0.367
	>25	51 (56.0)	52 (48.6)	
pN	≤5	42 (46.2)	58 (54.2)	0.324
	>5	49 (53.8)	49 (45.8)	
HER2	Negative	80 (87.9)	105 (98.1)	**0.009**
	Positive	11 (12.1)	2 (1.9)	
Lymphatic vessel invasion	No	48 (52.7)	60 (56.1)	0.745
	Yes	43 (47.3)	47 (43.9)	
Perineural invasion	No	49 (53.8)	75 (70.1)	**0.027**
	Yes	42 (46.2)	32 (29.9)	

Abbreviation: TLN, total lymph node; pN, positive lymph node; HER2, human epidermal growth factor receptor-2; LVI, lymphatic vessel invasion; PNI, perineural invasion. Bold values are primarily used to identify meaningful variables.

### The potential independent prognostic factors of DFS and OS for stage III gastric cancer patients who underwent curative resection

To further identify potential independent prognostic predictors of DFS and OS, we enrolled the common parameters into Cox proportional hazard regression model for univariate and multivariate analysis. The univariate and multivariate Cox regression analysis has shown that IOSS, PALB (prealbumin), TLN (total lymph node), tumor size, and postoperative chemotherapy were authenticated as potential independent factors to determine the DFS. Moreover, the IOSS, age, A/G (Albumin/Globulin), PALB (prealbumin), FIB (fibrinogen), TLN (total lymph node), and tumor size were authenticated as potential independent factors to determine the OS. The detailed information were shown in Table 4.

**TABLE 4:** The univariate and multivariate Cox proportional hazard regression analysis.

	DFS						OS					
	Univariate			Multivariate			Univariate			Multivariate		
Characteristics	HR	95% CI	*p*	HR	95% CI	*p*	HR	95% CI	*p*	HR	95% CI	*p*
IOSS	1.785	1.141–2.792	**0.011**	3.107	1.828–5.280	**0.000**	1.776	1.136–2.776	**0.012**	4.355	2.429–7.808	**0.000**
Sex	1.248	0.805–1.934	0.321				1.230	0.793–1.905	0.355			
Age	1.543	0.996–2.391	0.052				1.706	1.101–2.645	**0.017**	1.881	1.139–3.108	**0.01**4
BMI	1.197	0.779–1.840	0.412				1.195	0.778–1.838	0.416			
Alanine aminotransferase	0.814	0.530–1.250	0.347				0.789	0.514–1.211	0.278			
Aspartate aminotransferase	0.795	0.518–1.221	0.295				0.804	0.524–1.235	0.319			
γ-Glutamyl Transferase	0.903	0.588–1.386	0.640				0.903	0.589–1.386	0.642			
Tactate dehydrogenase	0.747	0.486–1.149	0.184				0.740	0.481–1.137	0.170			
Direct bilirubin	1.015	0.661–1.557	0.947				1.047	0.682–1.606	0.833			
Indirect bilirubin	0.786	0.512–1.209	0.273				0.769	0.500–1.181	0.230			
Total protein	1.211	0.789–1.858	0.382				1.216	0.792–1.866	0.372			
Albumin	0.974	0.633–1.497	0.903				0.980	0.638–1.507	0.928			
Globulin	1.550	0.994–2.415	0.053				1.499	0.962–2.335	0.074			
Albumin/Globulin (A/G)	0.538	0.348–0.831	0.005	0.745	0.442–1.254	0.268	0.553	0.358–0.853	0.007	0.690	0.406–1.171	0.169
Prealbumin	0.484	0.310–0.754	**0.001**	0.529	0.310–0.901	**0.019**	0.486	0.312–0.757	**0.001**	0.489	0.284–0.839	**0.009**
Blood urea nitrogen	1.261	0.818–1.943	0.294				1.273	0.826–1.962	0.275			
Alkaline phosphatase	0.978	0.637–1.500	0.919				1.071	0.698–1.643	0.754			
Glucose	1.024	0.667–1.574	0.912				1.048	0.683–1.610	0.829			
Cholesterol	1.080	0.704–1.658	0.724				1.095	0.714–1.680	0.678			
Triglyceride	1.106	0.721–1.698	0.644				1.134	0.739–1.741	0.565			
ABO blood type	1.150	0.914–1.446	0.232				1.149	0.914–1.445	0.234			
White blood cell	0.973	0.634–1.492	0.899				0.948	0.618–1.455	0.807			
Neutrophils	1.007	0.656–1.545	0.975				0.961	0.626–1.475	0.856			
Lymphocyte	0.833	0.543–1.279	0.405				0.865	0.564–1.328	0.507			
Monocyte	0.785	0.511–1.205	0.268				0.805	0.524–1.237	0.322			
Eosinophils	0.623	0.403–0.964	0.033	0.862	0.533–1.394	0.544	0.583	0.377–0.902	0.015	0.777	0.480–1.255	0.302
Basophil	0.900	0.586–1.381	0.628				0.883	0.575–1.355	0.568			
Hemoglobin	1.277	0.831–1.961	0.264				1.232	0.802–1.892	0.340			
Red blood cell	1.025	0.668–1.573	0.910				1.001	0.653–1.536	0.995			
Platelet	0.746	0.485–1.147	0.182				0.727	0.473–1.119	0.147			
International normalized ratio	1.214	0.789–1.869	0.378				1.246	0.809–1.917	0.318			
Fibrinogen	1.804	1.166–2.791	0.008	1.467	0.917–2.348	0.110	1.831	1.184–2.833	**0.007**	1.611	1.005–2.582	**0.048**
Carcinoembryonic antigen	0.862	0.561–1.324	0.499				0.888	0.578–1.364	0.587			
Alpha fetoprotein	0.853	0.556–1.308	0.466				0.838	0.546–1.286	0.418			
Carbohydrate antigen199	1.236	0.805–1.897	0.332				1.231	0.802–1.889	0.342			
Carbohydrate antigen724	1.066	0.695–1.635	0.770				1.069	0.697–1.64	0.761			
Radical resection	1.536	1.100–2.146	0.012	1.368	0.942–1.987	0.100	1.626	1.161–2.278	0.005	1.378	0.945–2.011	0.096
Type of surgery	1.214	0.937–1.573	0.141				1.298	1.003–1.681	0.048	1.295	0.968–1.734	0.082
Primary tumor site	1.118	0.836–1.496	0.452				1.076	0.800–1.447	0.628			
Borrmann type	1.332	0.988–1.795	0.060				1.308	0.977–1.751	0.071			
TLN	0.634	0.412–0.976	**0.038**	0.489	0.295–0.810	**0.005**	0.592	0.384–0.911	**0.017**	0.401	0.240–0.670	**0.000**
pN	1.160	0.756–1.780	0.496				1.099	0.717–1.686	0.664			
Tumor size	1.752	1.275–2.407	**0.001**	1.839	1.273–2.657	**0.001**	1.693	1.229–2.332	**0.001**	1.852	1.258–2.726	**0.002**
Differentiation	1.269	0.839–1.919	0.259				1.343	0.888–2.031	0.162			
Pathology	0.915	0.790–1.059	0.231				0.918	0.792–1.064	0.257			
pTNM stage	1.256	0.957–1.649	0.101				1.275	0.967–1.681	0.085			
pT stage	1.234	0.917–1.661	0.165				1.288	0.956–1.736	0.096			
pN stage	1.032	0.821–1.296	0.790				1.014	0.809–1.270	0.906			
Lauren type	0.895	0.697–1.149	0.383				0.883	0.686–1.138	0.338			
HER2	0.750	0.303–1.852	0.532				0.834	0.338–2.059	0.694			
Lymphatic vessel invasion	1.027	0.669–1.577	0.902				1.031	0.671–1.583	0.890			
Perineural invasion	2.464	1.136–5.346	0.022	1.341	0.595–3.020	0.479	2.393	1.103–5.191	0.027	1.306	0.581–2.936	0.519
Postoperative chemotherapy	1.654	1.067–2.564	**0.024**	1.587	1.003–2.510	**0.049**	1.726	1.113–2.677	0.015	1.469	0.910–2.373	0.116

Abbreviation: BMI, body mass index; TNM, tumor node metastasis; ALT, alanine aminotransferase; AST, aspartate aminotransferase; GGT, γ-glutamyl transferase; LDH, lactate dehydrogenase; TBIL, total bilirubin; DBIL, direct bilirubin; IDBIL, indirect bilirubin; TP, total protein; ALB, albumin; GLOB, globulin; PALB, prealbumin; A/G, ALB/GLOB; BUN, blood urea nitrogen; ALP, alkaline phosphatase; Glu, glucose; CHOL, cholesterol; TRIG, triglyceride; W, white blood cell; N, neutrophils; L, lymphocyte; M, monocyte; E, eosinophils; B, basophil; Hb, hemoglobin; R, red blood cell; P, platelet; INR, international normalized ratio; FIB, fibrinogen; CEA, carcinoembryonic antigen; AFP, alpha fetoprotein; CA199, Carbohydrate antigen199; CA724, Carbohydrate antigen724; TLN, total lymph node; pN, positive lymph node; HER2, human epidermal growth factor receptor-2; LVI, lymphatic vessel invasion; PNI, perineural invasion. Bold values are primarily used to identify meaningful variables.

### Association of IOSS with DFS and OS

**FIGURE 1:**
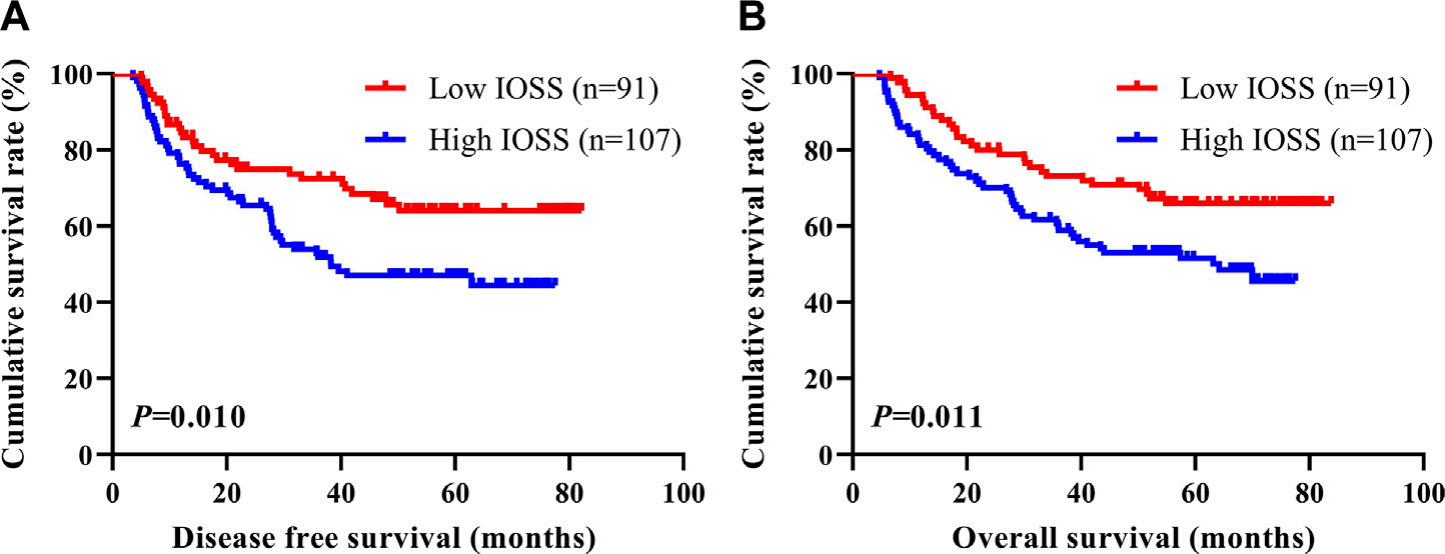
DFS and OS of patients with stage III gastric cancer. **(A)** Kaplan-Meier analysis of DFS for the IOSS, **(B)** Kaplan-Meier analysis of OS for the IOSS.

To determine the prognostic ability of IOSS in forecasting DFS and OS, the 198 stage III gastric cancer patients were separated into two groups: low IOSS ≤2.00 (N = 91) and high IOSS >2.00 (N = 107). The median DFS and OS in the low IOSS group were evidently longer than those in the high IOSS group (χ^2^ = 6.632, *p* = 0.010; χ^2^ = 6.519, *p* = 0.011). In terms of DFS, patients with low IOSS had higher 1-, 3-, and 5-year DFS rates [84.50% (95% CI: 0.773–0.923), 72.40% (95% CI: 0.636–0.825), and 64.1% (95% CI: 0.545–0.755) vs. 76.30% (95% CI: 0.686–0.849), 51.8% (95% CI: 0.428–0.626), and 46.9% (95% CI: 0.380–0.581)]. In terms of OS, patients with low IOSS had higher 1-, 3-, and 5-year OS rates [94.50% (95% CI: 0.899–0.993), 73.20% (95% CI: 0.645–0.830), and 65.9% (95% CI: 0.567–0.767) vs 81.30% (95% CI: 0.742–0.890), 59.8% (95% CI: 0.512–0.698), and 51.6% (95% CI: 0.428–0.622)]. Cumulative survival is shown in Figure 1.

### Nomogram development and validation

**FIGURE 2:**
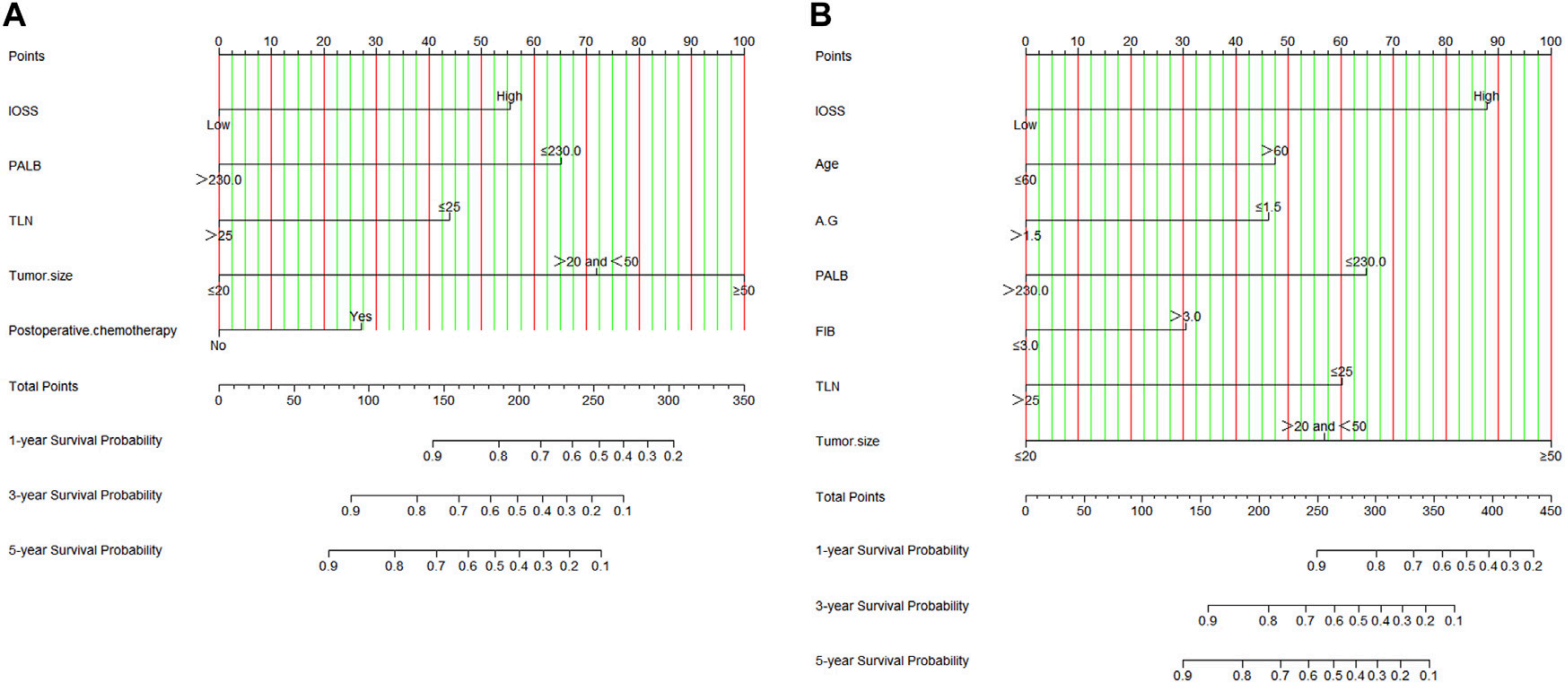
Nomograms by multivariate Cox proportional hazard regression analysis for predicting DFS and OS. **(A)** Nomogram for predicting DFS; **(B)** Nomogram for predicting OS.

**FIGURE 3:**
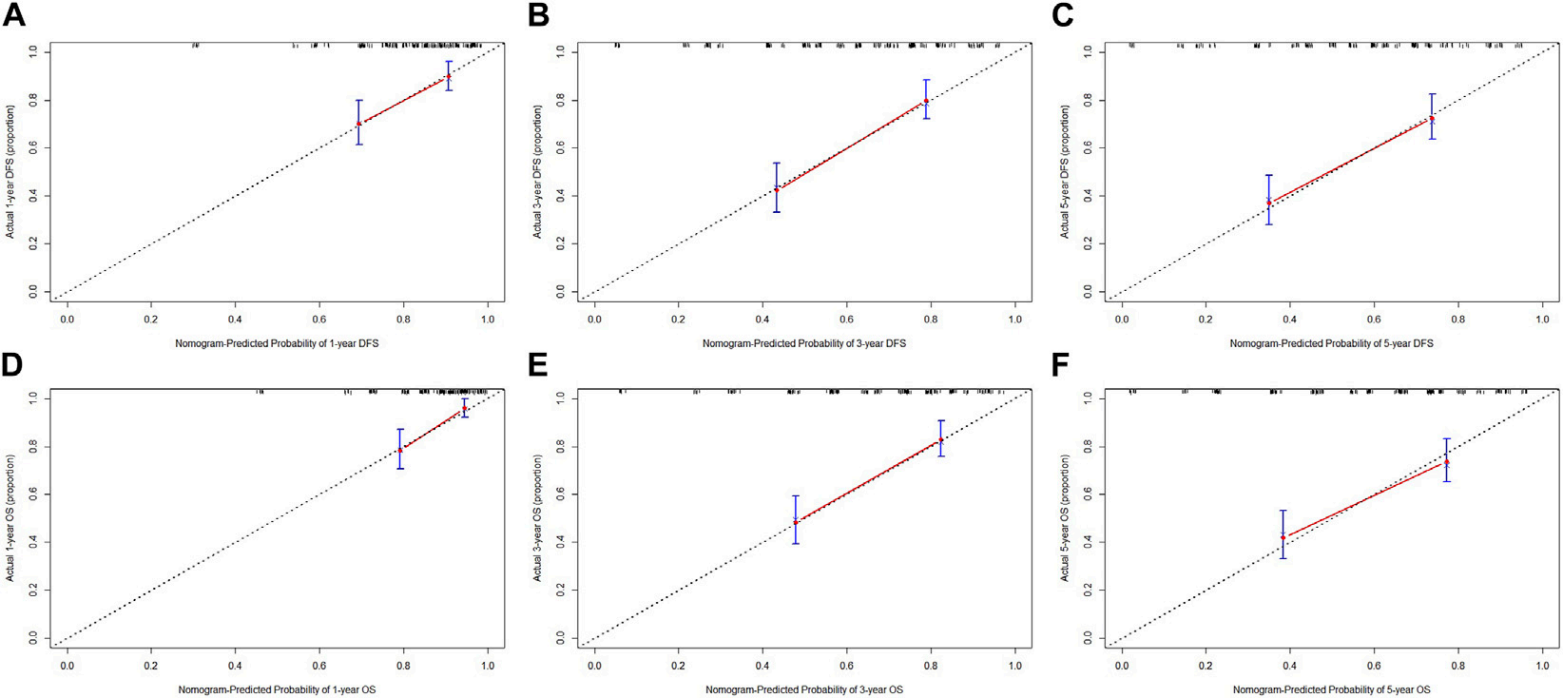
Calibration curve for evaluating the 1-, 3-, and 5-year DFS and OS rates. **(A)** Calibration curve for evaluating 1-year DFS rate; **(B)** Calibration curve for evaluating 3-year DFS rate; **(C)** Calibration curve for evaluating 5-year DFS rate; **(D)** Calibration curve for evaluating 1-year OS rate; **(E)** Calibration curve for evaluating 3-year OS rate; **(F)** Calibration curve for evaluating 5-year OS rate.

**FIGURE 4:**
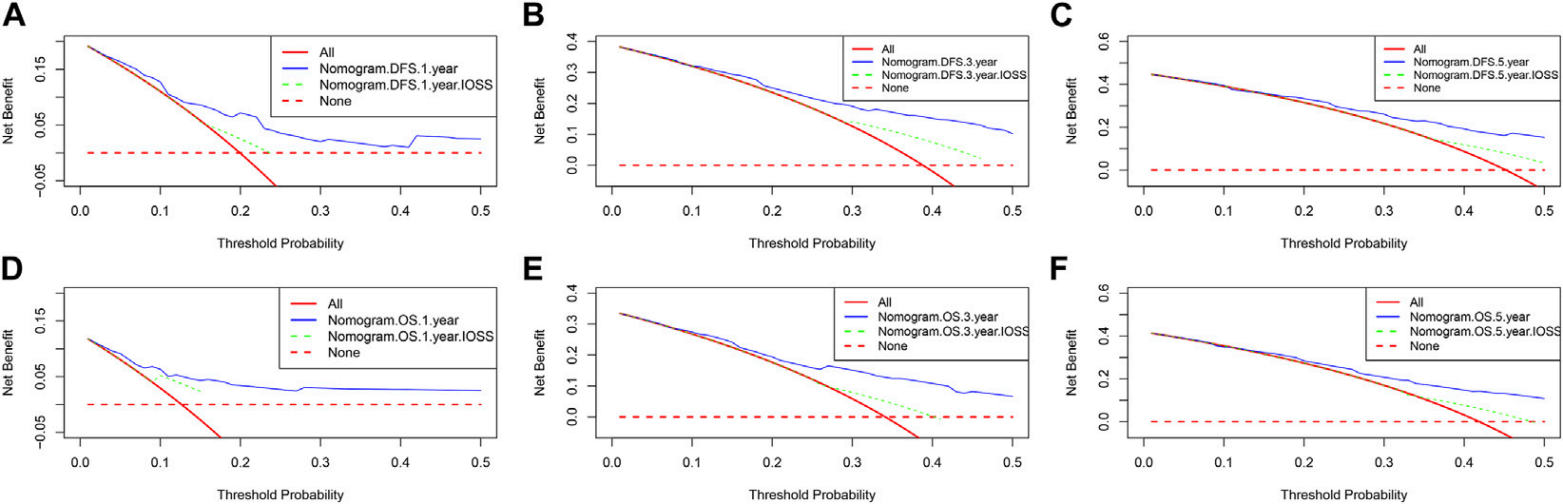
Decision curve analysis for the nomogram and only IOSS. **(A)** Decision curve analysis for 1-year DFS; **(B)** Decision curve analysis for 3-year DFS; **(C)** Decision curve analysis for 5-year DFS; **(D)** Decision curve analysis for 1-year OS; **(E)** Decision curve analysis for 3-year OS; **(F)** Decision curve analysis for 5-year OS.

Nomograms of the potential prognostic factors by the multivariate analysis for DFS and OS were established (Figure 2). The enrolled factors for DFS included IOSS, PALB (prealbumin), TLN (total lymph node), tumor size, and postoperative chemotherapy; and for OS included IOSS, age, A/G (Albumin/Globulin), PALB (prealbumin), FIB (fibrinogen), TLN (total lymph node), and tumor size via the multivariate analysis. Moreover, the calibration curve was performed to test predictive performance for DFS and OS after curative resection. The calibration curve has shown good agreement between predicted and actual probability at different survival time points (Figure 3). To further test predictive clinical utility, the decision curve analysis was applied to measure the net benefits under different threshold value probabilities. Compared to only IOSS, the constructed nomogram model (the nomogram incorporating the respective potential prognostic factors by the multivariate analysis) generated the best net benefit within the threshold probability for 1-, 3-, and 5-year survival time. Furthermore, the constructed nomogram model’s predictive clinical utility for clinical decision was better than IOSS (Figure 4).

## Discussion

Despite routine treatment with radical surgery and adjuvant therapy, the survival outcome remains unsatisfactory in gastric cancer, especially in stage III gastric cancer [23, 24]. According to the research report, half of gastric cancer patients will develop recurrence or metastasis within 5 years of systemic treatment [25]. At present, it is said that the damage of the antioxidant system will lead to the development of cancer, and the high oxidative stress status will increase risk of gastrointestinal malignancy [26]. Oxidative stress participates in the advancement of malignant tumors, including gastric cancer, however, it is not clear in gastric cancer prognosis [27, 28]. Therefore, accurate risk assessment is essential for supervising postoperative treatment and recurrence monitoring.

In the current research, we established a comprehensive score, IOSS, based on ALB (albumin), DBIL (direct bilirubin), and BUN (blood urea nitrogen), to predict the prognosis of stage III gastric cancer. We investigated the relationship between clinicopathological parameters and prognosis of gastric cancer. IOSS was related to age, radical resection, and tumor size. For elderly patients with gastric cancer, the probability of anemia, body mass decline, and malnutrition is high. The albumin is reflected by the nutritional status. Furthermore, the liver function is also affected by the gastric cancer disease, and the elderly patients with stage III gastric cancer are prone to liver dysfunction. The possibility of radical resection was low in patients with later TNM stage, and after surgery, the incidence rate of postoperative complications is high as well as poor nutritional status. According to univariate and multivariate Cox proportional hazard regression analysis, the result indicated that IOSS was a potential prognostic factor in gastric cancer patients. The prognostic value of IOSS was further analyzed, and indicated that patients with low IOSS survived longer, and with higher 1-, 3-, and 5-year survival rates. Moreover, the IOSS was significantly associated with age, radical resection, and tumor size. IOSS was also significantly associated with total protein (TP), albumin (ALB), prealbumin (PALB), blood urea nitrogen (BUN), triglyceride (TRIG), hemoglobin (Hb), and red blood cell (R). Furthermore, IOSS was significantly associated with HER2 and perineural invasion. These results indicated that oxidative stress had a critical influence on the prognosis in gastric cancer patients. Predicting metastasis and recurrence following curative resection is important for the management of stage III gastric cancer. At present, there are still no highly reliable and convenient biomarkers to predict metastasis and recurrence of gastric cancer.

In order to establish a pin-point accurate DFS and OS forecasting model, the potential prognostic factors from univariate analysis were taken a step further by multivariate analysis. The five valuable factors, IOSS, PALB (prealbumin), TLN (total lymph node), tumor size, and postoperative chemotherapy were used to conduct the predictive DFS model. The seven valuable factors, IOSS, age, A/G (Albumin/Globulin), PALB (prealbumin), FIB (fibrinogen), TLN (total lymph node), and tumor size were used to conduct the predictive OS model. The nomograms combine clinical characteristics from the multivariate analysis on DFS and OS to increase the accuracy of survival prediction, and were used to evaluate the prognosis in gastric cancer patients. The calibration curve has shown a good homogeneity between predicted and actual probability at different survival time points, including 1-, 3-, and 5-year survival rates. The decision curve analysis was used to quantify the net benefits under different threshold value probabilities, and the predictive clinical utility of the nomogram for clinical decision was better than IOSS.

Assessment of antioxidant administration and oxidative stress is very important for the prevention and treatment of gastric cancer [29–31]. The current study indicated that the oxidative stress was related to the initiation and development of gastric cancer. IOSS was a potential factor to improve the degree of accuracy of gastric cancer prognosis. Several plausible mechanisms are used to explain the association between IOSS and prognosis of stage III gastric cancer. Albumin is the most abundant plasma protein, accounting for one-half of the total protein content [32]. Low concentration of albumin may reflect a worse nutritional status, and the undernutrition may weaken the immune system and have an adverse impact on the prognosis of cancer patients [33]. Albumin is also an important factor in systemic inflammatory response [34]. Bile acid is the end-product of cholesterol decomposition, and consists of two forms in hepatocytes and peripheral blood, comprising direct bilirubin (DBIL) and indirect bilirubin (IDBIL) [35]. Serum bilirubin is the final product of blood metabolism, and has lots of barrier properties, including anticancer, antioxidant, and anti-inflammatory activities [36–38]. Furthermore, the bilirubin is negatively related to the risk of many of cancers, such as lung cancer, breast cancer, and gastrointestinal cancer [39–41]. Blood urea nitrogen (BUN) is manufactured by the metabolism of amino acids and protein in the body, and influences the physiological responses of human protein intake and metabolism [42]. Moreover, blood urea nitrogen (BUN) is a major factor reporting the association between nutritional status and renal status, and is associated with mortality [43].

A number of limitations for the current research should be considered. Firstly, this research was a retrospective single center study with a small number of patients. To go a step further to enrich the references, a prospective, multicenter, and well-designed study with more patients is needed. Secondly, multiple treatment measures were received by reason of tumor recurrence or metastasis during the follow-up periods, and might affect OS. The different postoperative treatments were not fully enrolled into the assessment of prognostic factors. Thirdly, even though the patients were chosen according to inclusion and exclusion criteria, selection bias was still inevitable. Finally, owing to the clinical record boundedness, the oxidative stress markers were not completely contained. Large-scale clinical research with more clinicopathological parameters carried out on multiple centers should be further studied to test and verify our findings.

## Conclusion

IOSS is a nonspecific tumor predictor based on procurable oxidative stress index, comprising ALB (albumin), DBIL (direct bilirubin), and BUN (blood urea nitrogen). Low IOSS is discovered to be a vigorous indicator of better prognosis. A nomogram conducted by combining the IOSS with other potential prognostic factors can predict the prognosis of gastric cancer patients.

## Data Availability

The raw data supporting the conclusion of this article will be made available by the authors, without undue reservation.
